# Shelf acetabuloplasty in children diagnosed with osteonecrosis of the femoral head secondary to acute lymphoblastic leukaemia

**DOI:** 10.1186/s12891-025-09362-9

**Published:** 2025-12-12

**Authors:** Ahmed El Ghoneimy, Ahmed Samy, Omneya Hassanain, Iman Zaki, Iman Sidhom, Hisham Abdel-Ghany

**Affiliations:** 1 Children cancer hospital, Cairo, Egypt; 2 Cairo University Hospitals, Cairo, Egypt

**Keywords:** Osteonecrosis hip, Avascular necrosis hip, Leukaemia, Leukaemia, Shelf osteotomy, Shelf acetabuloplasty

## Abstract

**Background:**

Shelf acetabuloplasty (SA) is a pelvic augmentation procedure often described in treatment of children diagnosed with Perthes disease of hip. This retrospective study is the first to report outcome in osteonecrosis of the femoral head (ONFH) secondary to acute lymphoblastic leukaemia (ALL).

**Patient and methods:**

All patients treated with SA in duration between 2017 and 2024 were included except for those who had follow-up less than one year following surgery. Patients selected for SA were those aged between 6 and 13-y with stage B or C collapse of the femoral head according to lateral pillar classification. The functional outcome was assessed pre-operative and at the latest follow-up using Harris Hip Score (HHS). The difference between both scores was compared using Wilcoxon Signed-Rank Test. Risk for advanced hip arthrosis was estimated based on Stulberg classification in radiograph done at latest follow-up.

**Results:**

A total of 15 patients were included: 7 males and 8 females. Four patients had bilateral hip involvement, with a total of 19 hips for evaluation. The mean age was 9.6 ± 2.19-y (6-13) at time of surgery and 14.1±2.70-y (10.3-18.4) at latest follow-up. Fifteen hips (79%) were Lateral Pillar stage B, and 4 (21%) were Lateral Pillar stage C. The mean follow-up duration was 44.7±21.00 (16-72) m. The median pre-operative HHS was 74 % (25, 42-88) and median post-operative HHS was 96% (20, 54-100), with a p-value of 0.002. According to Stulberg classification, 10 hips (52.6%) were grade 1 and 2 with no risk of hip arthritis, 5 (26.3%) grade 3 with mild risk, and 4 (20%) grade 4 and 5 with a high risk. Complications were graft resorption in two and infection in one hip.

**Conclusion:**

S.A. may improve coverage of the femoral head and the functional outcome of ALL patients diagnosed with advanced stage of ONFH and may potentially reduce the risk for advanced hip osteoarthrosis.

**Level of evidence:**

Retrospective case series, level IV.

## Introduction

Acute lymphoblastic leukaemia (ALL) is the most common paediatric cancer, accounting for a quarter of all malignancies diagnosed among children younger than 15 years of age. Dramatic advances in its treatment over the past decades have changed it from a universally fatal to an almost curable disease in more than 90% of cases [1]. Osteonecrosis of the femoral head (ONFH) is one of the reported side effects of chemotherapy in ALL, affecting up to 12% of patients [2] and its progression can lead to a significant long-term morbidity; 30% of patients with severe symptoms will require a total joint replacement surgery or will have long-term significant reduction in their quality of life [3]. Medications, physical therapy and percutaneous drilling are highly effective in most patients with early stage ONFH, but are most likely to fail in preventing the progression in advanced disease [4].

Shelf acetabuloplasty (SA) is a pelvic augmentation procedure in which an iliac crest bone graft augments the anterolateral part of the acetabulum to provide coverage for the femoral head. It was described in Perthes disease of the hip [5, 6] and in ONFH secondary to sickle disease [7] to prevent the lateral subluxation of the femoral head and hinge abduction and to minimise the risk for progression of the femoral head deformity and hip osteoarthritis. Although the pathogenesis of Perthes disease may differ from drug induced ONFH of the femoral head in children, the radiologic changes of the femoral head described in both conditions are similar, and the extrusion of the necrotic femoral head plays an important role in natural history of both diseases [8, 9] In our institute, SA was adopted in selected cases of ONFH in ALL patients since 2017 and till current times, and this is the first report in literature to describe the outcome in this specific patients’ cohort. The primary objective was to compare the radiologic and functional outcome before and after SA and to assess the risk for advanced osteoarthrosis of the hip at final follow-up. The secondary objective was to describe complications following surgery, their management and outcome.

## Patient and methods

### Clinical setting

The study was conducted in the orthopaedic department of a paediatric cancer institute. We used the lateral pillar classification described by Herring [10] to define our strategy for managing patients diagnosed with ONFH. Before 2017, the treatment strategy for patients with ONFH and Lateral pillar stage A was a combination of medical treatment (pain medications, calcium and vitamin D), percutaneous drilling and protected weight bearing and for those with Lateral Pillar stage B and C was improving function and quality of life by physical therapy ± soft tissue release when needed. Following 2017, we began to treat stage B and C patients surgically by SA and percutaneous drilling, provided that the patient chronological age was between 6 and 13-y at the time of surgery; patients < 6-y were considered to have a high remodelling potential of their femoral head and would improve if treated conservatively while patients > 13-y were considered to have a limited remodelling potential of the femoral head not sufficient enough to allow for the desired adaptive remodelling following surgery.

### Surgical technique

The timing of surgery was discussed priorly with oncologist and chemotherapy was halted 7–10 days before and following surgery. A complete blood picture with a minimum Hb of 9 gm/dl, WBCs of 1000/µL and platelets of 150,000/µL was required before surgery. In a supine position, 3 drill-holes were performed, percutaneously and under fluoroscopic visualisation, using a smooth 3 mm K-wire to decompress the femoral epiphyses. In 11 hips (58%) with palpable adductor tightness, a medial release of the adductor longus was performed through a medial transverse incision. Shelf acetabuloplasty was then performed using the previously described Spitzy technique [11]: a curvilinear incision extended from middle of the iliac crest downward and medially 2 cm inferior to the anterior superior iliac spine. The iliac apophysis was stripped off the crest and reflected together with the attached glutei away from the outer table of the ilium by subperiosteal dissection downward until reaching the reflected head of rectus femoris muscle and the joint capsule. An iliopsoas release was performed by an intramuscular tenotomy done through the medial end of the incision. Under fluoroscopic visualisation, the exact acetabular edge was identified, and a bone trough was performed just above the subchondral bone of the acetabulum at its anterolateral aspect by sharp osteotome. A bi-cortical bone graft, 20 × 20 mm, was harvested from the exposed iliac crest and impacted in the bone trough so that 10 mm of the graft were left protruding over the capsule and the anterolateral part of the femoral head. The bone graft was fixed by k-wires (1–2 wires) and/or a buttressing 3.5 reconstruction plate according to the surgeon’s discretion. Post-operative, the hip was immobilized in a short hip spica for 3–4 weeks.

### Inclusion and exclusion criteria

Following the approval of the IRB, our longitudinally maintained electronic record system (REDCap) was screened for patients diagnosed with ONFH secondary to ALL and treated by SA in duration between 2017 and 2024. *Inclusion criteria*: 1- Patients age between 6 and 13 years at diagnosis, 2- A radiograph demonstrating a Lateral pillar stage B or C collapse of the femoral head. *Exclusion criteria*: 1- Lateral pillar stage A, 2- Follow-up less than one year due to death from disease or patients lost to follow-up before completing one year following surgery.

### Data collection and outcome measures

All pre, intra and post-operative clinical and radiographic data were collected from a longitudinally kept electronic health record system (Oracle Cerner,2022) including the stage of ONFH (Ficat and Arlet classification) at pre-operative and final follow-up (Table 1), the Lateral pillar classification based on which patients were selected for SA, the Centre Edge angle (CE) reflecting the coverage of the femoral head in the pre-operative and final radiographs, and finally the function of the hip pre-operative and at final follow-up using Harris Hip Score (HHS) [12]. The score was described in an ordinal scale: scores between 90 and 100 were categorized as Excellent, 80–89 as Good, 70–79 as Fair, and < 70 as Poor. The prognosis for late development of severe hip osteoarthrosis was radiographically categorized, at the latest follow-up visit, using Stulberg classification [9, 13].

**Table 1:** Patient demographics, stage of ONFH, disease interval and prior management before surgery

	Gender	Age(ys)	ALL Risk stratification*	Interval between diagnosis ONFH and surgery (months)	Ficat and Arlet (pre-operative)	Ficat and Arlet (final FP)	Conservative measures	FP period (months)
1	M	6	Low	8	2b	1	Physical therapy, calcium and vit D	59
2	F	7	Intermediate	8	2b	1	Calcium and vit D	48
3	F	8	Intermediate	22	3	3	Physical therapy, calcium and vit D	35
4	M	9	Intermediate	22	3	3	Physical therapy, calcium and vit D	72
5	M	9	Intermediate	30	3	3	Physical therapy, calcium and vit D	65
6	M	12	Intermediate	2	2b	3	none	60
7	M	11	Intermediate	7	3	1	Physical therapy, calcium and vit D	72
8	F	11	Intermediate	0	3	3	none	65
9	M	10	High	6	2b	1	Physical therapy, calcium and vit D	57
10	F	8	Intermediate	2	3	3	none	36
11	M	12	Intermediate	9	2b	1	Calcium and vit D	66
12	F	12	Intermediate	3	3	3	Core decompression, physical therapy, calcium and vit D	20
13	F	13	Intermediate	8	3	3	Core decompression, physical therapy, calcium and vit D	16
14	F	6	Intermediate	1	2b	1	Physical therapy	66
15	M	9	Intermediate	0	3	3	none	18
16	F	9	High	5	2b	1	Calcium and vit D	30
17	F	9	High	8	2b	1	Calcium and Vit D	27
18	F	10	High	2	2b	2b	Physical therapy, calcium and vit D	20
19	F	13	Intermediate	0	2b	3	none	19

*All patients received the same chemotherapeutic drugs (vincristine, doxorubicin, asparaginase cyclophosphamide, mercaptopurine, cytarabine and methotrexate) according to St. Jude total therapy XV protocol which is followed in our institution

### Statistical methods

All statistical analyses were performed using SPSS (version 20, 2011). Variation in continuous variables were described using mean and standard deviation or median and interquartile range and categorical variables using measures of frequency and proportions. A Wilcoxon Signed-Rank Test was conducted to compare difference between CE pre-operative and at final follow-up at the diseased hip and to compare difference between CE of the normal hip and the diseased hip at final follow-up in the 11 patients with unilateral involvement. We also compared Harris Hip Score (HHS) before and after surgery, with a p-value < 0.05 considered as statistically significant.

## Results

A total of 18 patients with diagnosis of ONFH were treated by SA. Three patients were excluded from the analysis due short follow-up (death of disease less than one year following surgery), leaving a final of 15 patients,7 males and 8 females. Among these, 4 patients had a bilateral hip involvement, with a total of 19 hips for evaluation. The mean age was 9.6 ± 2.19-y (6–13) at the time of surgery and 14.1 ± 2.70-y (10.3–18.4) at latest follow-up. Table 1 lists stage of ONFH according to Ficat and Arlet, any prior treatment to SA, and interval between diagnosis and shelf procedure. Fifteen hips (79%) were Lateral Pillar stage B, and 4 (21%) were Lateral Pillar stage C. The mean follow-up duration was 44.7 ± 21.00 m. (16–72).

### Primary outcomes

**Fig. 1:**
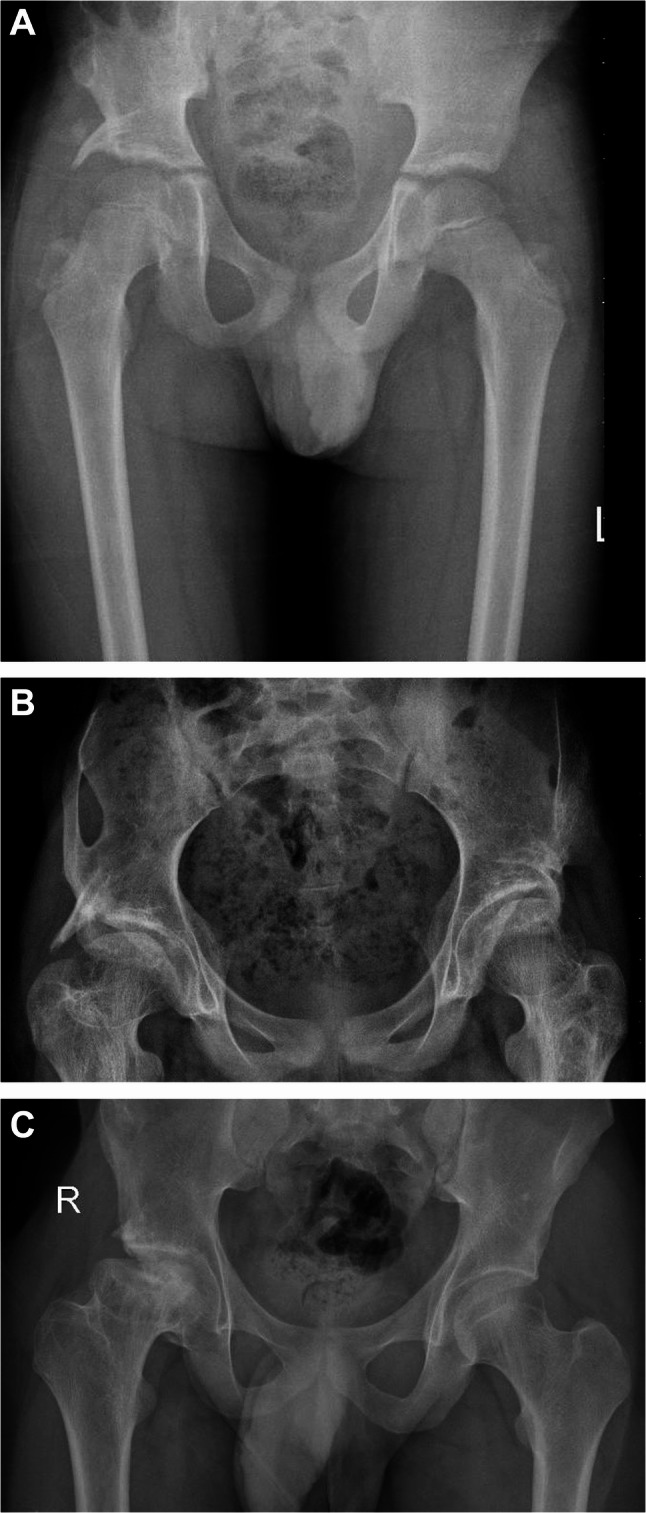
**A** A male patient, diagnosed with an ONFH Lateral Pillar grade B at the age of 6-y. Radiograph done at the age of 11-y, 59-m following SA, demonstrating a grade- 1 Stulberg of the right hip. **B** A female patientdiagnosed with bilateral ONFH lateral pillar grade B at the age 8-y. Radiograph done at the age of 11.9-y, 48-m following SA, demonstrating a grade- 3 Stulberg of the right hip and grade-2 of the left hip. **C** A male patient, diagnosed with ONFH Lateral Pillar grade B at the age of 13-y. Radiograph done at the age of 17-y, 60-m following SA, demonstrating grade-5 Stulberg of the right hip

The median CE was 20° (13, 5–47) and 29° (14, 11–54) in the pre-operative and final radiographs of the diseased side, respectively, with a statistically significant difference (p-value, 0.025). The median CE of the normal side was 26 (10,23–38) with no statistically significant difference between it and CE of the diseased side at final follow-up (p-value,0.623). The median HHS was 74% (25, 42–88) and 96% (20, 54–100) pre-operatively and at final follow-up, respectively. Pre-operative, 7 patients had a Good, 6 had a Fair and 6 had a Poor functional score. Post-operative, 12 patients had an Excellent, 1 had a good, 3 had a fair and 3 had a poor functional score. Using Wilcoxon Rank-sign test, there was a statistically significant difference between pre-operative and final HHS (*p*-value, 0.002). According to Stulberg classification, 10 hips (52.6%) were grade 1 and 2 with good prognosis (Fig. 1A), 5 (26.3%) grade 3 with mild risk for osteoarthrosis (Fig. 1B), and 4 (20%) grade 4 and 5 with a high risk for advanced osteoarthrosis (Fig. 1C), at their latest follow-up. In two out of four hips with grade 4 or 5 Stulberg classification, a severe hip pain and limitation of movement was observed during follow-up. Both patients had a total hip replacement at 19 and 21 m. following SA. at an age of 14.5-y and 15-y.

### Secondary outcomes

**Fig. 2:**
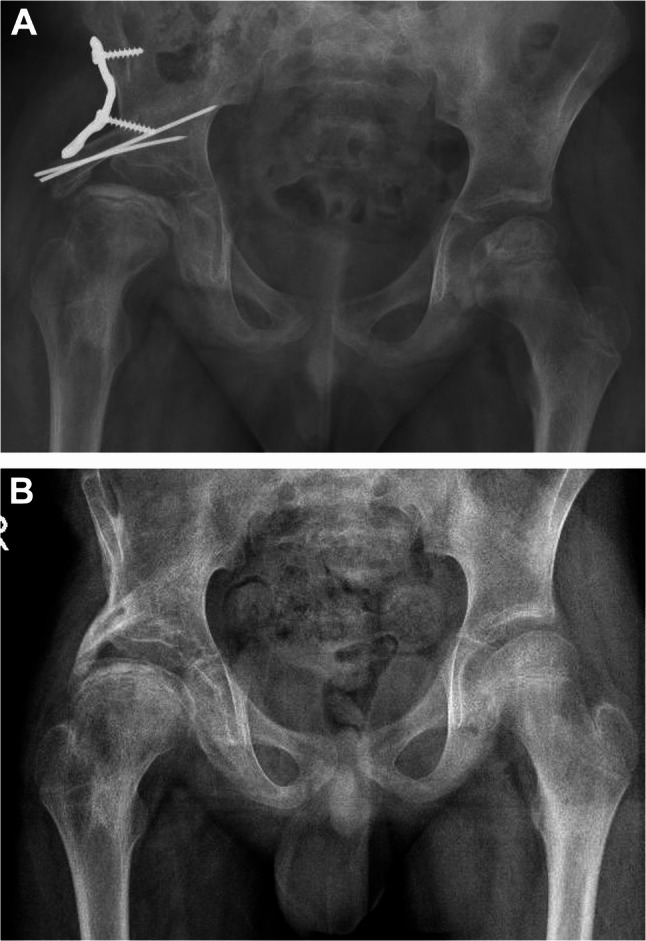
**A** A male patient diagnosed with a right hip ONFH lateral pillar grade C at the age of 9.5-y. Immediate post-operative radiograph following SA. **B** Radiograph after the removal of reconstruction plate and wires 18-m following SA, demonstrating a healed shelf graft and grade 3 Stulberg of the right hip

A total of 3 complications were observed in 3 out of 19 hips (16%): graft resorption in two and post-operative wound infection in one hip. Graft resorption was managed conservatively by continued follow-up for evidence of progression. In one hip, necrosis progressed to advanced osteoarthrosis of the hip and the patient had a poor HHS at her latest follow-up which was managed by a total hip replacement 21 months following SA. In the other hip, no progression was observed, and the patient had a good HHS and Stulberg grade 3 hip at his latest follow-up. In the case of infection, by the time of diagnosis the shelf graft was already healed, and the infection was controlled following the removal of the reconstruction plate. At the latest follow-up, the patient had an excellent HHS and Stulberg grade 2 hip (Fig. 2A-C).

## Discussion

The routine screening of all leukaemia patients under treatment for ONFH using MRI is not a standardized practice, and, consequently, the reported incidence and severity varies considerably between different cancer centres [4]. A considerable number of patients may have advanced radiographic findings at the time of diagnosis for which medical treatment and percutaneous drilling may not succeed in halting the progression of osteonecrosis [14]. In our experience, adding SA to percutaneous drilling of the femoral head led to the improvement in the functional outcome and in the prognosis for advanced osteoarthrosis of the hip when done in patients with Lateral pillar B or C collapse of the femoral head.

In the previously described non-operative and operative treatment strategies, favourable outcomes were limited to early stages (pre-collapse) of the disease. In a retrospective study on 80 ALL patients with ONFH, Karimova et al. investigated the influence of several risk factors on joint collapse including previous core decompression. The size of the lesion was the only significant independent variable for joint collapse irrespective of whether a core decompression was done or not. They stated that among the 35 core decompressions they performed for patients with collapsed articular surface and those with large sized lesions, they did not observe any evidence that core decompression prevented or delayed joint collapse or arthroplasty [15]. Hyperbaric oxygen was tried by Biddeci et al. in 8 out of 23 children diagnosed with ONFH. Three patients had early necrosis (ARCO grade 1 and 2) and 4 had advanced stage (ARCO grade 3 and 4). The lesion was stable in the early lesions, but all 4 patients with advanced necrosis progressed in follow-up MRI and underwent joint arthroplasty [14]. This was contrary to what we observed following SA which we performed only in advanced stages of ONFH and, at the latest follow-up, 80% of the patients had a low risk for advanced osteoarthrosis of their hips.

Previous studies described the outcome of SA in other hip disorders in children such as Perthes disease of the hip [5, 6, 16]. Vinayak et al. operated on 35 patients with Perthes disease of the hip and, most of their patients had an excellent and good HHS and were Stulberg 1 to 3 at their latest follow-up. They concluded that shelf procedure results in favourable clinical function and saves the congruency of the hip [17, 18]. In another study, SA have also been shown not to complicate future total hip replacement in progressed cases with advanced osteoarthrosis of the hip [19].

Controversies exist in literature regarding the technique: some advocate bi-cortical versus uni-cortical iliac crest bone graft and some use internal fixation of graft while others do not [20]. In our cohort we used internal fixation of bi-cortical grafts. Bi-cortical bone grafts are easier to fix with wires or plates. Internal fixation serves to prevent proximal migration of graft which was reported in some series [16].

A major limitation was the lack of a control group for comparison due to the incomplete data on the patients treated before 2017 in our electronic record system. The inability to compare patients treated conservatively with those treated by SA prevents exclusion of the contribution of natural disease regression or quiescence to HHS improvements. The small sample size, due to rarity of the condition, limits the statistical power of the analyses and conclusions must be cautiously generalized. finally, the HHS is a subjective score filled out by the physician and the authors were not blinded during assessment of Stulberg grade in the follow-up radiographs which subjects the study to a considerable selection bias.

In conclusion, preliminary data suggest that S.A. may improve coverage of the femoral head, and the functional outcome of ALL patients diagnosed with advanced stage of ONFH, potentially reducing the risk of advanced hip osteoarthrosis.

## Data Availability

all excel data, statistical analysis and radiographs are available upon request without any patient identifier included.
